# Supplementary material for: Weed community changes in saffron+chickpea intercropping under different irrigation management
Source: PLoS One. 2023 May 26;18(5):e0286474. doi: 10.1371/journal.pone.0286474 (PMC10218734; doi:10.1371/journal.pone.0286474)
Supplement: Attachment — Submitted filename: Associate editor comments.docx [file pone.0286474.s002.docx]

Comments and suggestions

- Manuscript English needs to improve.
- Provide some quantitative data in the abstract for a better understanding.
- Provide details such as the number of saffron plants in mono-cropping, nutrient management, and other agronomic practices followed in this experiment, what was the size of the main and subplot in this study.
- Provide the test names for normality and homogeneity of variance in the statistics section.
- Add crop observations, economics, and yield data in the result section to strengthen the manuscript.
- For more comments please see the manuscript.
