# Supplementary material for: Weed community changes in saffron+chickpea intercropping under different irrigation management
Source: PLoS One. 2023 May 26;18(5):e0286474. doi: 10.1371/journal.pone.0286474 (PMC10218734; doi:10.1371/journal.pone.0286474)
Supplement: Attachment — Submitted filename: Response to the reviewers comment.docx [file pone.0286474.s003.docx]

**Dear Dr. ....,**

We would like to thank you for the letter, and the opportunity to submit a revised copy of this manuscript. We would also like to take this opportunity to express our thanks to the reviewers for the comments. We believe have resulted in an improved revised manuscript, which you will find uploaded alongside this document. The manuscript has been revised to address the reviewer's comments, detailed in your letter properly as explained below.

ACADEMIC EDITOR:

1- Manuscript English needs to improve.

- It was corrected

2- Provide some quantitative data in the abstract for a better understanding.

- It was added in abstract.

3- Provide details such as the number of saffron plants in mono-cropping, nutrient management, and other agronomic practices followed in this experiment, what was the size of the main and subplot in this study.

- It was added in M&M section.

4- Why not before 75 days

- Because before 75 days, weeds had not been completely established in the plots. The aim of evaluating the weed biomass and weed density was estimation the effective role in decreasing saffron and chickpea yield then we decided to measure the weed traits after completely establishing them.

5-You followed the wrong practice

- It was corrected

6- Provide the test names for normality and homogeneity of variance in the statistics section.

- The data were first checked for normal distribution and variance homogeneity using the Shapiro–Wilk test (PROC UNIVARIATE) and the Bartlett test (PROC GLM), respectively. Weed density (plant m^-2^) and biomass (g m^-2^) were square-rooted and log-transformed, respectively.

7- Add crop observations, economics, and yield data in the result section to strengthen the manuscript.

- Because this article was part of a doctoral dissertation and crop observations, economics, and yield data were used in the previous article then we decided to cite to that article here.
- It was cited in the article in lines 251-256.
- The crop observations, economics, and yield data were indicated in the other extracted reports from this excremen which indicated that, the effect of the irrigation regime was not significant on flower number, fresh weight of flower, and dried stigma yield of saffron. In intercropping ratios, the highest flower number (141.5 ± 4.26 m^−2^), fresh weight of flower (108.22 ± 3.4 g m^−2^), and dried stigma yield (4.1 ± 0.01 kg h^−1^) were observed in saffron mono-crop and approximately equaled by C3 treatment (61).
- Mohammadkhani, F., Pouryousef, M. and Yousefi, A.R., 2023. Growth and production response in saffron-chickpea intercropping under different irrigation regimes. *Industrial Crops and Products*, *193*, p.116256.

8- Need to mention also the appropriate ratio

- It was added in the abstract

9- Provide some data in the abstract for better understanding

- It was added in the abstract

10-You followed the wrong practice

- In was corrected.

11- Please ensure that you refer to Table 4 in your text as, if accepted, production will need this reference to link the reader to the Table.

- I really appreciate your nice comment. We referred to Tables 3 and 4 in the result and discussion section, and the following sentences were added.
- Based on the results of combine analysis of the effect of irrigation regimes (I), and intercropping system (C) on weed control and weed diversity properties in Table 3 (164-172 lines).

Reviewer #1:

Weed community changes in saffron–chickpea intercropping under different irrigation management-This research investigated irrigation regimes and intercropping, and their effect on weed control and diversity. The research is unique and would be of interest to PLOS ONE readers.

a. The title is confusing looking like you used only an intercrop of saffron and chickpea. But in this study, you also used sole saffron and sole chickpea.
The English and scientific writing needs improvement.

- In was corrected

b. There are several details missing to fully describe material & methods effectively: how do you calculate the net plot in this study?

- It was added in lines 128-130.

c. Nutrient management part is missing.

- It was added in lines 130-132.

d. What was the nutrient content in studied soil?

- It was added in table 2.

e. Size of each main plot?

- This note was added in line 128 to 130.

f. You collected weed data at 75 DAS of chickpea. But by this time the weeds would have affected the saffron.

- Because before 75 days, weeds had been not completely established in the plots. The aim of evaluating the weed biomass and weed density was estimation the effective role in decreasing saffron and chickpea yield then we decided to measure the weed traits after completely establishing them.

g. How you checked normality and variance homogeneity of data?

- The data were first checked for normal distribution and variance homogeneity using the Shapiro–Wilk test (PROC UNIVARIATE) and the Bartlett test (PROC GLM), respectively. Weed density (plant m^-2^) and biomass (g m^-2^) were square-rooted and log-transformed, respectively.

h. What was the objective to take sole chickpea here?

- Sole chickpea crop stand for monoculture of chickpea.

i. Follow the journal (PLOS ONE) pattern in all references.

- All references were by Mendeley and was based on journal(PLOS ONE) pattern

j. Italic all scientific names.

- Thank you, the text was edited.

Specific comments;

1 Full and short titles both are the same.

- It was corrected.

2 Arrange keywords in alphabetical order.

- It was corrected.

3 Line 17: Both in eight rows. What does it mean?

- It is refer to eight row of saffron plants.

4 Line 36: Kg ha-1 make it in superscript.

- It was corrected.

5 Line 37: Delete production.

- It was corrected.

6 Line 50: This sentence needs a complete revision to make it sound good.

- It was corrected.

7 Line 72: Vicia faba in italic.

- It was corrected.

8 Line 80-82: Remove too much result based references from introduction.

- It was corrected.

9 Line 83: Add space in between "%" and "27".

- It was corrected.

10 Line 85: Cicer arietinum in italic.

- It was corrected.

11 Line 93: Delete according to.

- It was corrected.

12 Line 94: could be in place of be could.

- It was corrected.

13 Line120: plant m-2 in place of plant m2.

- It was corrected.

14 Line 125: How was the sample location selected in each plot?

- For sampling; in each plot an area was selected after ignoring 0.5 m from each side of the plots.

15 Line132 and 133: Eh and Ln are change in formula.

- It was corrected.

16 Line239: Resource in place of resource.

- It was corrected.

17 Line 254: et al?

- It was corrected.

18 The discussion part is poor and lacks proper explanation and reasoning.

- It was corrected.

19 To make their writing easier to read, the authors should improve it.

- It was corrected.

Reviewer #2:

I have gone through the manuscript "Weed community changes in saffron–chickpea intercropping under different irrigation management". There study was carried out at semi-arid cold ecologies of Zanjan, Iran in the randomized block design. The manuscript explains an environment-friendly system of weed control. Considering the novelty, lack of information on saffron+chickpea intercropping system and the quality of MS, I feel that there are several strong reasons to accept the MS after a moderate level of revisions.

Addressing the below points will add more value to the MS:
Questions to be addressed

I. What is the effect of this system on saffron yield?

- The crop observations, economics, and yield data were indicated in the other extracted reports from this excremen which indicated that, the effect of the irrigation regime was not significant on flower number, fresh weight of flower, and dried stigma yield of saffron. In intercropping ratios, the highest flower number (141.5 ± 4.26 m^−2^), fresh weight of flower (108.22 ± 3.4 g m^−2^), and dried stigma yield (4.1 ± 0.01 kg h^−1^) were observed in saffron mono-crop and approximately equaled by C3 treatment (61).

II. What about the cost-effectiveness of this system (net profits and cost: benefit ratio)?

- According to our results, the saffron yield parameters were not affected by a one-time irrigation regime, which indicated that saffron could be a proper option in arid and semiarid regions. Among intercropping treatments, C3 treatment had the same effects as saffron mono-crop on the saffron. Therefore, C3 treatment could be substituted for saffron mono-crop.

III. How the crop management was done and what agronomic management was practiced. Corrections: Line 83 - %, Line 94 – could be, Line 239 – resource

- It was corrected in M&M

   IV. Write saffron + chickpea instead of saffron - chickpea as "+" is more appropriate and internationally acceptable for intercropping systems.
- It was corrected in M&M

V. The results in the abstract need to be written in quantitative terms. Merely writing statements are not sufficient for a scientific paper.

- It was corrected.

VI. The discussion part is poorly written. Please discuss the results in a more comprehensive manner.

- It was corrected.

**Best regards,**

**Corresponding author,**
