# Supplementary material for: Weed community changes in saffron+chickpea intercropping under different irrigation management
Source: PLoS One. 2023 May 26;18(5):e0286474. doi: 10.1371/journal.pone.0286474 (PMC10218734; doi:10.1371/journal.pone.0286474)
Supplement: Attachment — Submitted filename: Manuscript revision (clean copy) .docx [file pone.0286474.s004.docx]

**Weed community changes in saffron–chickpea intercropping under different irrigation management**

Fatemeh Mohammadkhani^1^, Majid Pouryousef^1*^, Ali Reza Yousefi^1^, Jose L. Gonzalez-Andujar^2^

^1^ Department of Plant Production & Genetics, University of Zanjan, Postal code: 4537138791, Zanjan, Iran;

^2^ Instituto de Agricultura Sostenible (CSIC), Avda. Menendez Pidal s/n, 14040 Córdoba, Spain.

^*^Corresponding author E-mail: pouryousef@znu.ac.ir

**Abstract**

Saffron (*Crocus sativus* L.) is one of the most expensive crops worldwide; however, it is a poor competitor to weeds. Intercropping and reduced irrigation are non-chemical farming practices to decrease weed problems. Therefore, the present study aimed to evaluate the changes in the weed density, biomass and weed diversity under saffron-chickpea intercropping system with two irrigation regimes. The treatments were two irrigation regimes [i.e. one-time irrigation and conventional irrigation (four occasions from October through May)] and six planting ratios of saffron and chickpea [i.e. saffron sole-crop (C1), chick­­­­­­­­pea sole-crop (C2) both in eight rows, 1:1 (C3), 2:2 (C4), 2:1 (C5), and 3:1 (C6)] as main and sub-plots, respectively. Result showed that the conventional irrigation regimes increased weed diversity, however, it didn't affect the Pielou index. Intercropping ratios decreased weed diversity compared to saffron and chickpea mono-cropping systems. The interaction effect of treatments was significant for weed density and weed biomass. In most intercropping ratios, weed density and weed biomass decreased under one-time irrigation regimes. The lowest value for weed density and weed biomass with an average 15.5 plant.m^2^ and 37.51 g.m^2^ were observed for one-time irrigation with C4 intercropping systems, this intercropping system was comparable with C3. Overall, the results indicated that a one-time irrigation regime and intercropping with chickpea with chick­­­­­­­­pea mono-crop and (C3) 1saffron:1chickpea, (C4) ratio could be the proper strategies for weed management in saffron in semiarid cropping systems.

**Keywords:** Biodiversity**,** Pielou index, Shannon index, Species richness, Weed biomass, , Weed control, ,

**Introduction**

Saffron (*Crocus sativus L*.) is one of the most expensive spice, medicinal and industrial crops worldwide (1). The plant is used as a spice to flavor food, and fragrance in perfumes and cosmetics worldwide. It has been used in traditional medicine as an anti-depressant, anti-carcinogen, and stimulant, along with a wide range of other applications (2, 3). The main saffron-producing countries are Iran, India, Greece, Afghanistan, Morocco, Spain, Italy, China, and Azerbaijan (4). Iran is the leading country in saffron production (5), with 120,000 ha, and 3.2 kg ha^−1^ as cultivated area, and average yield, respectively (6). Iran produces almost 90% of global, saffron(7).

Saffron is cultivated in different climatic conditions, for example, in southern Europe, North Africa, the Middle East, and Central Asia, which have low rainfall, warm summers, and cold winters (8). It has a lower water requirement than many other crops due to its morpho-physiological properties (9) and drought tolerance and having a dormant stage (from May to August) (10). Nevertheless, irrigation increases saffron yield; and is therefore irrigated four or five times from October through May (11). De Juan, Córcoles (12) reported that irrigation in March and April, as well as in August and September, increases saffron flower and stigma yield. However, Koocheki and Seyyedi (13) indicated that in western Macedonia irrigation increased leaf development, but reduced the number of flowers and quality of saffron.

After flowering in October, saffron grows actively through April and then the plant senesces, leaves wither (April−May), and corms become dormant approximately until July. Therefore, the fields are without any crop canopy during this time, and weeds can occupy the space and suppress the saffron plant. Therefore, weeds are the main problem in saffron production, since this plant is a poor competitor with weeds and grows slowly, so weed growth at any stage of crop growth can hurt the quality and quantity of the product. Weeds are controlled by mechanical, hand weeding, or chemical methods. Although mechanical and hand weeding are effective and environmentally friendly, they are time-consuming and costly (14, 15). Hosseini-Evari et al. (16) revealed that a combination of herbicide (Metribuzin) with the mulch method can be useful for weed management in saffron. Chemical weed control is too expensive and has negative side effects on the environment and human health (17).

Irrigation management is one of the strategies of weed control for the development of a weed management system, especially in arid and semiarid regions. Therefore, a low irrigation schedule during the weed life cycle could reduce weed germination and presence by less water availability for weed growth. Methvaly et al (18) stated that in peanuts, total weed dry weight reduced up to 25.66–25.71% because of decreased irrigation by 60%. Verma et al. (19) reported that weed density and biomass were reduced at the lower rate of irrigation because of the less availability of water.

Another strategy for preventing weed dominance is using intercropping systems. This cultivation system, the simultaneous cultivation of one or multiple crop species in the same field area, is a good practice to enhance productivity through efficient use of nutrients, light, and water while suppressing weeds (20-23). Moghaddam et al. (24) showed that saffron-white bean intercropping, concurrent with 10 mM glycine betaine application, can play a pivotal role in regulating soil temperature and increasing land equivalent ratio. Some evidence showed that intercropping can decrease weeds' germination, emergence, and flowering (25-27). Shabahang et al. (28) reported that *Vicia faba* with saffron intercropping decreased weed density by 93% as compared to weed-infested plots. Shah et al. (29) showed that bean-rice intercropping reduced weed presence by 65%. Saudy (30) reported that maize–cowpea intercropping suppressed weeds over all N levels, reducing weed biomass by 49.5% compared to sole-maize. Bilalis et al. (31) pointed out that intercropping maize with legumes significantly decreased weed density compared with mono-cropping corn by reducing light access for weeds.

Research on the effects of intercropping on weed community structure (or diversity) is limited. Intercropping can change weed community structure (32-34), mainly due to the increasing emergence of the spring species (34). Berdjour et al., (35) reported that intercropping corn-soybean decreased weed species diversity by 40% compared to the sole-crop. Sharma & Banik, (36) showed a weed diversity reduction by the intercropping system.

Legumes represent about % 27 and % 15 primary agricultural production and proteins worldwide, respectively, (37). Legumes are considered a key species in intercropping systems due to nitrogen fixation. In this study, chickpea (*Cicer arietinum* L.) was used as the companion crop. Chickpea, a small annual legume from the Fabaceae family, is the third most cultivated legume in the world dry bean (Phaseolus vulgaris) and peas (Pisum sativum) (38). The plant is mostly planted in arid and semi-arid regions and has an important role in intercropping systems because it adds nitrogen to the soil (39), reduces chemical inputs, and improves soil fertility (40, 41). Abbasi and Sepaskhah (42) reported that farmers can use the inter-row spaces of the saffron crop to grow winter wheat simultaneously in order to obtain higher revenue and land productivity, and make clean and sustainable production.

saffron planted in arid and semiarid regions, and is a poor weed competitor, resource limitation like water, and the application of intercropping systems could be effectively in weed control saffron fields. To the best of our knowledge, there is no study on the effect of the saffron–chickpea intercropping system on weed populations. Therefore, the present research aimed to study the effects of saffron–chickpea intercropping on weed control and community structure (or diversity) under two irrigation regimes.

**Material and methods**

***Site description***

The present study was performed for two growing seasons from 2018-19 to 2019-20 at the Research Farm of the Faculty of Agriculture, University of Zanjan (latitude 36° 41′ N, longitude 48° 27′ E; 1620 m above sea level), Zanjan, Iran. The climate of this region is classified as semi-arid cold, with an average annual temperature of 11 °C and average annual precipitation of 293 mm (Fig. 1). The soil characteristics of the experimental site were as follows: sandy-loam texture, 7.54 pH, and 1.1% organic matter.

***Experimental design and treatments***

The field experiments were arranged in a split-plot pattern with three replications. Each plot was 2m × 2m in size.

In mono-cropping, saffron was planted with a distance of 25 cm between the rows and 7 cm between the plants on the row and at a depth of 15-20 cm, which was a total of 80 plant/m2. Chickpeas were also cultivated with a density of 50 plants/m2 with a depth of 3 to 4 cm.

The first factor included two drip irrigation regimes i.e. one-time irrigation and conventional irrigation as main plots which the size of each main plot was 14.5×2 m and size of subplots was 2m × 2m. Before planting, a disk and leveler were used to prepare the land for cultivation, sowing data was in the middle of the summer of 2017, after preparing the land and preparing the seedbed, 30 tons of rotted cattle manure was applied to the land and mixed completely with the soil.

The one-time irrigation treatment was applied following saffron planting in October, and conventional irrigation was applied on four occasions from October through May according to rainfall conditions (Table 1). The second factor consisted of six planting ratios of saffron: chickpea, (C1) saffron mono-crop, (C2) chick­­­­­­­­pea mono-crop, (C3) 1saffron+1chickpea, (C4) 2saffron+2chickpea, (C5) 2saffron+1chickpea, and (C6) 3saffron+1chickpea; details are presented in Figure 2. Saffron corms and chickpea seeds were planted manually in 25-cm row spacing (80 and 50 plant m-2, respectively), in early October and March 2018, respectively (Fig. 2). No herbicides or pesticides were applied throughout the experiment. Also, chemical and physical soil characteristics was indicated in table 2.

***Measurements and sampling***

Aboveground weeds were hand collected from 1 m^2^ area in each subplot at 75 days after sowing (DAS) of the chickpea in 2019 and 2020. Weed species were identified and counted based on the photosynthetic system. Weed biomass was oven-dried at 63 °C for 72h and dry weights (g m^-2^) were determined. For sampling, in each plot were selected area after ignoring 0.5 m from each sides of plots.

To assess weed diversity, Species richness (S), the Exponential Shannon Index, and Pielou’s Evenness Index were calculated for each plot and year separately**.** Species richness (S) is the weed species' total number. the Exponential Shannon Index (eH; (43), was estimated as :

$Eh=exp[-\sum_{i=1} pi Ln (pi)]$

Where Eh is the exponential Shannon Index, pi is the relative abundance of the i species in the community, and Ln is the natural logarithm.

Pielou’s Evenness Index (J′; (44) was calculated as;

$J^{'}=[\frac{H^{´}}{{H´}_{Max}}]$

Where J′ is Pielou’s Evenness Index, H is the number derived from the Shannon diversity index and H′ max is the maximum possible value of H’ (if every species was equally present).

***Statistical analysis***

Statistical analysis was performed using PROC GLMMIX in SAS (version 9.4). The data were first checked for normal distribution and variance homogeneity using the Shapiro–Wilk test (PROC UNIVARIATE) and the Bartlett test (PROC GLM), respectively. Weed density (plant m^-2^) and biomass (g m^-2^) were square rooted and log-transformed, respectively. Mean comparisons were done using the least significant difference (LSD) test at a 95% confidence level (P ≤ 0.05). Because there was a significant effect of year and there were several treatment interactions data were analyzed and presented separately by the year.

**Results and discussion**

Based on the results of combine analysis of the effect of irrigation regimes (I), and intercropping system (C) on weed control and weed diversity properties in Table 3, the main effect of year were significant at 5 and 1 % probability for Exponential (Shannon Index) and Species richness, respectively. Also, the main effect of irrigation were significant at 1, 1, 5, and 5 % probability for Weed density, Weed biomass, Exponential (Shannon Index) and Species richness respectively. The mine effect of intercropping was the same as irrigation, the only different between that is Pielou Index was significant at 5% probability in this effect. About interaction effect of IR*CR weed density and weed biomass were significant at 5 % probability level (Table 3)**.**

***Weed flora***

A total of nine weed species were identified during both years of the experiment, and only two species had a perennial life cycle. Two weed species belonging to [Poaceae](https://en.wikipedia.org/wiki/Poaceae) families, had a C_4_ photosynthetic pathway, whereas the other remaining species had a C_3_ photosynthetic pathway (Table 4).

***Weed density and weed biomass response to irrigation and intercropping***

The application of one-time irrigation significantly reduced total weed density compared to conventional irrigation. A similar pattern was observed in the weed biomass, where minimum weed biomass with an average 37.51 g m^-2^ was obtained by the one-time irrigation regime and C4 intercropping system. Therefore, the one-time irrigation regime provided greater weed control. According to the results, intercropping reduced both weed density and weed biomass compared to the saffron and chickpea mono-crops (C1 and C2). The treatment C4 (2:2 ratio) and C3 (1:1 ratio), were effective in reducing weed density and weed biomass. The interaction of irrigation regimes and intercropping systems showed that one-time irrigation with strategy C4 provides more control of weeds in terms of weed density and weed biomass and obtained with an average 37.51 and 15.5 respectively (Fig. 3A, 3B). In summary, the results indicated that the one-time irrigation regime and the intercropping system (strategy C4) can effectively control weeds.

Crop management practices shape the weed community, influencing the productive performance of the crops. Among these practices, irrigation is a major factor influencing weed flora composition, especially in semi-arid regions. In line with our results, other studies have also reported that the one-time irrigation treatment was more effective in reducing weed density and biomass (45). The superiority of the one-time irrigation regime as a weed control tool in comparison to conventional irrigation can be explained by a reduction in weed emergences as a consequence of less water availability during the weed life cycle.

The changes in water availability during the germination period of weed affect the seedling emergence pattern, reducing weed density and biomass (46). Verma, Singh (19, 47) reported that low irrigation decreased *Chenopodium album* biomass due to an insufficient supply of water. ud Din, Ramzan (48) reported higher density and biomass values for *Rumex Crispus*, *Convolvulus Arvensis*, and *Ammi Visnaga* L*.* in plots with 100% irrigation.

Intercropping treatments in both irrigation regimes reduced weed density and biomass compared to mono-cropping. Some previous studies agree with our findings (49, 50). Weed density and biomass reductions can be attributed to higher solar radiation intercepted by the canopy of crops in the intercropping system, therefore reducing light availability for weeds (31). Katsaruware (51) reported that intercropping maize with cowpea has decreased weed biomass as compared to sole-crop, and has limited the accessibility of resources for weeds such as PAR reaching the ground. Eskandari (52) stated that intercropping of wheat with fava-bean was more effective to control weeds compared to the wheat mono-crop. Banik, Midya (39) reported that wheat–chickpea intercropping could decrease weed density and biomass by 70%. Szumigalski and Van Acker (53) showed greater weed suppression in the intercropping system compared to their mono-crop in wheat-canola and wheat-canola- pea intercrop. ***Weed diversity*** ***response to irrigation and intercropping***

According to the results, the conventional irrigation increased the weed diversity in the plots compared to the one-time irrigation (Fig. 4). There were no significant differences between the two irrigation regimes in terms of the Pielou index (equity, Fig. 4). The intercropping treatments reduced the weed diversity compared to the mono-crop treatments (C1 and C2 strategies). Species richness was reduced by C3 and C4 systems (Fig. 5).

There were no significant differences between the treatments in terms of the Pielou index in the 2020 crop season. The C5 and C6 systems showed a significant reduction in the Pielou index compared to the monoculture systems during the 2019 crop season. The C4 treatment significantly reduced the exponential Shannon index in both years, compared to the saffron and chickpea mono-crop treatments (Fig. 5). A significant difference was observed between years in terms of the e(Shannon index) and species richness, where the maximum stated values were recorded during the second year (Fig. 6).

In agricultural systems with less water availability, the dominance of weeds more adapted to water stress conditions may intensify competition, reducing agrosystems' diversity (54). Higher levels of weed species richness and diversity (exponential Shannon index) have been found under the conventional irrigation treatment compared to the one-time irrigation. Some studies have stated that the once water supply restricts the establishment of species that are not tolerant to drought stress conditions (55-57). Nevertheless, the evenness index was similar in both irrigation systems. As the degree of evenness in plant communities is mediated by competitive interactions (58), our results suggested that no large differences have arisen in weed composition. It has been shown by different researchers that intercropping alters the structure of weed communities (32, 34).

A significant difference between years in terms of the e(Shannon index) and species richness might be due to relatively higher precipitation and less soil temperature during this year. The species richness and diversity were found to be lower in the intercropping treatments compared to the mono-cropping treatments. One reason for this phenomenon could be the competitive advantage of the crop plants over weeds in the intercropping system due to higher solar radiation interception by the crop plants and reduced light availability for weeds.

Widaryanto (59) found that a decreased light intensity received by weeds due to intercropping reduced the diversity of species in the weed communities. Baumann et al. (60) reported that intercropping of leeks increased weed suppression due to increased light absorption by vegetation, which ultimately reduced the number of weeds.

Differences in the species richness of intercropping systems over experiments can be demonstrated by capturing light available from the intercropping system canopy; as a result, light availability is reduced and causes weed suppression and weed diversity reduction. Similar results were reported in previous studies (31, 33, 36).

The crop observations, economics, and yield data was indicated in the other extracted reports from these excrements which indicated that, the effect of the irrigation regime was not significant on flower number, fresh weight of flower, and dried stigma yield of saffron. In intercropping ratios, the highest flower number (141.5 ± 4.26 m^−2^), fresh weight of flower (108.22 ± 3.4 g m^−2^), and dried stigma yield (4.1 ± 0.01 kg h^−1^) were observed in saffron mono-crop and approximately equaled by C3 treatment (61).

Based on these results, which reported in previous article (61) and weed controlling by intercropping system in saffron, and chickpea it could be related to the nitrogen fixation by chickpea as a leguminous crops and saffron as economical crop which produce the suitable yield in this cropping system.

**Conclusion**

According to the result, low irrigation regimes had a significant effect on weed density andbiomass as well as on weed diversity. Low irrigation regimes reduced the density, biomass, and diversity of weeds as compared to the conventional irrigation regimes through limiting the water availability to weeds. In addition, the intercropping system showed a significant effect on weed density andbiomass, and weed diversity in both irrigation regimes. Intercropping systems reduced weed density and biomass as compared to mono-crop. The weed diversity was reduced with saffron-chickpea intercropping. by limiting the recourse availability such as water, light, etc. . Consequently, low irrigation regimes and intercropping systems could be a proper strategy for weed control in the saffron field, especially in arid and semiarid regions.

**Acknowledgement**

This research received no specific grant from any funding agency or the commercial or not-for-profit sectors.

**Conflict of interest**

No conflicts of interest have been declared.

**References**

1.	Leone S, Recinella L, Chiavaroli A, Orlando G, Ferrante C, Leporini L, et al. Phytotherapic use of the Crocus sativus L.(Saffron) and its potential applications: A brief overview. Phytotherapy Research. 2018;32(12):2364-75.

2.	Pandita D. Saffron (Crocus sativus L.): Phytochemistry, therapeutic significance and omics-based biology. Medicinal and Aromatic Plants: Elsevier; 2021. p. 325-96.

3.	Jan S, Wani AA, Kamili AN, Kashtwari M. Distribution, chemical composition and medicinal importance of saffron (Crocus sativus L.). African Journal of Plant Science. 2014;8(12):537-45.

4.	Shahnoushi N, Abolhassani L, Kavakebi V, Reed M, Saghaian S. Economic analysis of saffron production. Saffron: Elsevier; 2020. p. 337-56.

5.	Dastranj M, Sepaskhah AR. Saffron response to irrigation regime, salinity and planting method. Scientia Horticulturae. 2019;251:215-24.

6.	Koocheki A, Moghaddam PR, Seyyedi SM. Saffron-pumpkin/watermelon: A clean and sustainable strategy for increasing economic land equivalent ratio under limited irrigation. Journal of Cleaner Production. 2019;208:1327-38.

7.	Bazrafshan O, Etedali HR, Moshizi ZGN, Shamili M. Virtual water trade and water footprint accounting of Saffron production in Iran. Agricultural water management. 2019;213:368-74.

8.	Rezvani-Moghaddam P. Ecophysiology of saffron. Saffron: Elsevier; 2020. p. 119-37.

9.	Behdani M, Fallahi H. Saffron: Technical knowledge based on research approaches. University of Birjand Publication, Birjand, IR (In Persian). 2015.

10.	Kafi M, Koocheki A, Rashed M. Saffron (Crocus sativus): production and processing: Science Publishers; 2006.

11.	Sepaskhah A, Kamgar-Haghighi A. Saffron Irrigation Regime. International Journal of Plant Production. 2009;3:1.

12.	de Juan JA, Córcoles HL, Muñoz RM, Picornell MR. Yield and yield components of saffron under different cropping systems. Industrial Crops and Products. 2009;30(2):212-9.

13.	Koocheki A, Seyyedi SM. Effects of different water supply and corm planting density on crocin, picrocrocin and safranal, nitrogen uptake and water use efficiency of saffron grown in semi-arid region. Notulae Scientia Biologicae. 2016;8(3):334-41.

14.	Kumar R, Singh V, Devi K, Sharma M, Singh M, Ahuja PS. State of art of saffron (Crocus sativus L.) agronomy: A comprehensive review. Food Reviews International. 2008;25(1):44-85.

15.	Mzabri I, Rimani M, Charif K, Kouddane N, Berrichi A. Effect of Shade on Agro-Morphological Parameters and Weed Flora of Saffron (Crocus sativus L.) Cultivation in the Semiarid Zone of Eastern Morocco. The Scientific World Journal. 2022;2022.

16.	Hosseini-Evari Z, Izadi-Darbandi E, Kafi M, Makarian H. Effect of Organic Mulch with Pre-emergence Herbicide Combinations on Weeds Control and Saffron Yield. 2022.

17.	Pavlović D, Vrbničanin S, Anđelković A, Božić D, Rajković M, Malidža G. Non-Chemical Weed Control for Plant Health and Environment: Ecological Integrated Weed Management (EIWM). Agronomy. 2022;12(5):1091.

18.	El-Metwally I, Abido W, Saadoon S, Gad S. The integrated effect of deficit irrigation and weed control treatments on peanut productivity under sandy soil conditions with reference to nematode infection. Plant Archives. 2020;20(1):2581-93.

19.	Verma S, Singh RP, Kumar S. Effects of irrigation and herbicides on the growth, yield and yield attributes of wheat (Triticum aestivum L.). Bangladesh Journal of Botany. 2017;46(3):839-45.

20.	Mastinu A, Kumar A, Maccarinelli G, Bonini SA, Premoli M, Aria F, et al. Zeolite clinoptilolite: Therapeutic virtues of an ancient mineral. Molecules. 2019;24(8):1517.

21.	Huang C, Liu Q, Gou F, Li X, Zhang C, van der Werf W, et al. Plant growth patterns in a tripartite strip relay intercrop are shaped by asymmetric aboveground competition. Field Crops Research. 2017;201:41-51.

22.	George O. Intercropping and its application to banana production in East Africa: A review. Journal of plant breeding and crop science. 2009;1(2):013-5.

23.	Vandermeer JH. The ecology of intercropping: Cambridge university press; 1992.

24.	Moghaddam PR, Seyyedi SM, Banhangi FM. The possible positive effect of white bean intercropped with saffron under glycine betaine application and limited moisture. Scientia Horticulturae. 2022;304:111300.

25.	Ramesh K, Rao A, Chauhan BS. Role of crop competition in managing weeds in rice, wheat, and maize in India: A review. Crop protection. 2017;95:14-21.

26.	Weerarathne L, Marambe B, Chauhan BS. Does intercropping play a role in alleviating weeds in cassava as a non-chemical tool of weed management?–A review. Crop protection. 2017;95:81-8.

27.	Weerarathne L, Marambe B, Chauhan BS. Intercropping as an effective component of integrated weed management in tropical root and tuber crops: A review. Crop protection. 2017;95:89-100.

28.	Shabahang J, Khorramdel S, AMIN GA, GHESHM R. Effects on management of crop residues and cover crop planting on density and population of weeds and agronomical characteristics of saffron (Crocus sativus L.). 2013.

29.	Shah TM, Tasawwar S, Bhat MA, Otterpohl R. Intercropping in rice farming under the system of rice intensification—An agroecological strategy for weed control, better yield, increased returns, and social–ecological sustainability. Agronomy. 2021;11(5):1010.

30.	Saudy HS. Maize–cowpea intercropping as an ecological approach for nitrogen-use rationalization and weed suppression. Archives of Agronomy and Soil Science. 2015;61(1):1-14.

31.	Bilalis D, Papastylianou P, Konstantas A, Patsiali S, Karkanis A, Efthimiadou A. Weed-suppressive effects of maize–legume intercropping in organic farming. International Journal of Pest Management. 2010;56(2):173-81.

32.	Mesgaran MB, Mashhadi HR, Khosravi M, Zand E, Mohammad-Alizadeh H. Weed community response to saffron–black zira intercropping. Weed science. 2008;56(3):400-7.

33.	Poggio SL. Structure of weed communities occurring in monoculture and intercropping of field pea and barley. Agriculture, ecosystems & environment. 2005;109(1-2):48-58.

34.	Poggio SL, Satorre EH, de la Fuente EB. Structure of weed communities occurring in pea and wheat crops in the Rolling Pampa (Argentina). Agriculture, ecosystems & environment. 2004;103(1):225-35.

35.	Berdjour A, Dugje IY, Dzomeku IK, Rahman NA. Maize–soybean intercropping effect on yield productivity, weed control and diversity in northern Ghana. Wiley Online Library, 2020 1444-6162.

36.	Sharma RC, Banik P. Baby corn-legumes intercropping system: II weed dynamics and community structure. NJAS-Wageningen Journal of Life Sciences. 2013;67:11-8.

37.	Meena RS, Yadav RS, Meena H, Kumar S, Meena YK, Singh A. Towards the current need to enhance legume productivity and soil sustainability worldwide: a book review. 2015.

38.	Acevedo Martinez KA, Yang MM, Gonzalez de Mejia E. Technological properties of chickpea (Cicer arietinum): Production of snacks and health benefits related to type‐2 diabetes. Comprehensive Reviews in Food Science and Food Safety. 2021;20(4):3762-87.

39.	Banik P, Midya A, Sarkar B, Ghose S. Wheat and chickpea intercropping systems in an additive series experiment: advantages and weed smothering. European Journal of agronomy. 2006;24(4):325-32.

40.	Leonetti P, Accotto GP, Hanafy MS, Pantaleo V. Viruses and phytoparasitic nematodes of Cicer arietinum L.: biotechnological approaches in interaction studies and for sustainable control. Frontiers in plant science. 2018;9:319.

41.	Hiremath PJ, Farmer A, Cannon SB, Woodward J, Kudapa H, Tuteja R, et al. Large‐scale transcriptome analysis in chickpea (Cicer arietinum L.), an orphan legume crop of the semi‐arid tropics of Asia and Africa. Plant biotechnology journal. 2011;9(8):922-31.

42.	Abbasi MR, Sepaskhah AR. Evaluation of Saffron Yield Affected by Intercropping with Winter Wheat, Soil Fertilizers and Irrigation Regimes in a Semi-arid Region. International Journal of Plant Production. 2022:1-19.

43.	Weaver W, Shannon C. The mathematical theory of communication University of Illinois Press Urbana. 1963.

44.	Pielou EC. An introduction to mathematical ecology. An introduction to mathematical ecology. 1969.

45.	Singh R, Singh B. Effect of irrigation time and weed management practices on weeds and wheat yield. Indian Journal of Weed Science. 2004;36(1-2):25-7.

46.	Rühl AT, Eckstein RL, Otte A, Donath TW. Distinct germination response of endangered and common arable weeds to reduced water potential. Plant Biology. 2016;18:83-90.

47.	Verma S, Singh S, Prasad S, Meena R, Meena R. Influence of irrigation regimes and weed management practices on water use and nutrient uptake in wheat (Triticum aestivum L. Emend. Fiori and Paol.). Bangladesh Journal of Botany. 2015;44(3):437-42.

48.	ud Din S, Ramzan M, Khan R, Rahman MU, Haroon M, Khan TA, et al. Impact of tillage and mulching practices on weed biomass and yield components of maize under rainfed condition. Pakistan Journal of Weed Science Research. 2013;19(2).

49.	Susha V, Das T, Nath C, Pandey R, Paul S, Ghosh S. Impacts of tillage and herbicide mixture on weed interference, agronomic productivity and profitability of a maize–Wheat system in the North-western Indo-Gangetic Plains. Field crops research. 2018;219:180-91.

50.	Saucke H, Ackermann K. Weed suppression in mixed cropped grain peas and false flax (Camelina sativa). Weed Research. 2006;46(6):453-61.

51.	Katsaruware R. Maize-cowpea intercropping and weed suppression in leaf stripped and detasselled maize in Zimbabwe. 2009.

52.	Eskandari H. Intercropping of wheat (Triticum aestivum) and bean (Vicia faba): Effects of complementarity and competition of intercrop components in resource consumption on dry matter production and weed growth. African Journal of Biotechnology. 2011;10(77):17755-62.

53.	Szumigalski A, Van Acker R. Weed suppression and crop production in annual intercrops. Weed Science. 2005;53(6):813-25.

54.	Wood SA, Karp DS, DeClerck F, Kremen C, Naeem S, Palm CA. Functional traits in agriculture: agrobiodiversity and ecosystem services. Trends in ecology & evolution. 2015;30(9):531-9.

55.	Sojneková M, Chytrý M. From arable land to species-rich semi-natural grasslands: Succession in abandoned fields in a dry region of central Europe. Ecological Engineering. 2015;77:373-81.

56.	Chauhan BS, Manalil S, Florentine S, Jha P. Germination ecology of Chloris truncata and its implication for weed management. PLoS One. 2018;13(7):e0199949.

57.	Burke IC, Thomas WE, Spears JF, Wilcut JW. Influence of environmental factors on after-ripened crowfootgrass (Dactyloctenium aegyptium) seed germination. Weed Science. 2003;51(3):342-7.

58.	Wilsey B, Stirling G. Species richness and evenness respond in a different manner to propagule density in developing prairie microcosm communities. Plant ecology. 2007;190(2):259-73.

59.	Widaryanto E. Weed communities on monoculture and intercropping cultivation techniques. Journal of Degraded and Mining Lands Management. 2017;4(3):781.

60.	Baumann DT, Bastiaans L, Kropff MJ. Competition and crop performance in a leek–celery intercropping system. Crop Science. 2001;41(3):764-74.

61. Mohammadkhani, F., Pouryousef, M. and Yousefi, A.R., 2023. Growth and production response in saffron-chickpea intercropping under different irrigation regimes. Industrial Crops and Products, 193, p.116256.

**Table 1.** Water quantity and the time of application

| Quantity of water applied (M^3^ ha^-1^) | Forth irrigation | Third irrigation | Second irrigation | First irrigation | Irrigation regimes |
| --- | --- | --- | --- | --- | --- |
| 700 | - | - | - | Early-October | One-time irrigation |
| 2800 | Mid-May | Late-April | Early-March | Early-October | Conventional irrigation |

Table 2. Physical and chemical properties of farm soil

| Organic Carbon (%) | Total nitrogen (%) | Phosphorus (ppm) | Potassium (ppm) | pH | EC (ds/m) | Soil texture |
| --- | --- | --- | --- | --- | --- | --- |
| 0.85 | 0.092 | 13.4 | 283 | 7.94 | - | Clay loam |

**Table 3.** Combine analysis of the effect of irrigation regimes (I), and intercropping system (C) on weed control and weed diversity properties.

| S.O.V | Df | MS | | | | |
| --- | --- | --- | --- | --- | --- | --- |
|  |  | Weed density | Weed biomass | Exponential (Shannon Index) | Pielou Index | Species richness |
| Year(Y)  Repeat(Y) | 1  4 | 0.0043^ns^  0.1782 | 0.0003^ns^  0.0020 | 0.0990*  0.0438 | 0.0051^ns^  0.0077 | 37.5555**  0.9722 |
| Irrigation(IR)  Y*IR  Repeat*IR(Y) | 1  1  4 | 1.6200**  0.0150^ns^  0.0236 | 0.0690**  0.0000^ns^  0.0010 | 0.3667*  0.0049^ns^  0.0100 | 0.0042^ns^  0.0007^ns^  0.0033 | 22.2222*  2.0000^ns^  0.1944 |
| Intercropping(CR)  IR*CR  Y*CR  Y*IR*CR  Error | 5  5  5  5  40 | 2.3118**  0.5203*  0.0346^ns^  0.1134^ns^  0.0014 | 0.0543**  0.0267*  0.0006^ns^  0.0006^ns^  0.0813 | 0.1279*  0.0114^ns^  0.0018^ns^  0.0170^ns^  0.0182 | 0.0051*  0.0009^ns^  0.0014^ns^  0.0022^ns^  0.0018 | 7.6222*  1.3555^ns^  0.4888^ns^  0.4000^ns^  0.4000 |

Ns, not significant; *, **: significant at P≤0.05 and 0.01, respectively.

**Table 4.** Weed flora composition of the experimental site.

| Photosynthetic pathway | Life Cycle | Family | Common name | Weed species |
| --- | --- | --- | --- | --- |
| C3 | Perennial | Convolvulaceae | Bindweed, Field | *Convolvulus arvensis* L. |
| C3 | Annual | Chenopodiaceae | Lambs quarters, Common | *Chenopodium album* L. |
| C3 | Annual | Polygonaceae | Knotweed, Prostrate | *Polygonum aviculare* L |
| C3 | Annual | Brassicaceae | Flixweed, Herb-sophia | *Descurainia sophia* L. |
| C3 | Annual | Convolvulaceae | Field dodder, Golden dodder | *Cuscuta compestris* Yunck |
| C3 | Annual | Polygonaceae | Dock, CurlY | *Rumex crispus* L. |
| C3 | Annual | Brassicaceae | Garlic | *Alyssum hirsutum* M. Bieb |
| C4 | Perennial | Poaceae | Bermuda grass | *Cynodon dactylon* L. |
| C4 | Annual | Poaceae | Cockspur, Barnyard millet | *Echinochloa crus-galli* L. |

**Fig. 1.** Average daily soil temperature (^o^C) at 9 cm and precipitation in 2019 (A) and 2020 (B). Meteorological data from Zanjan University Meteorological Station (from 2018-19 through 2019-20), available in (http://www.znu.ac.ir/weather)

**Fig. 2.** Schematic diagram of saffron and chickpea intercropping based on row replacement series. (C1) saffron mono-crop, (C2) chick­­­­­­­­pea mono-crop, (C3) 1saffron:1chickpea, (C4) 2saffron:2chickpea, (C5) 2saffron:1chickpea, and (C6) 3saffron:1chickpea, respectively. Saffron corms and chickpea seeds were planted with 25-cm row spacing.

**Fig.3.** Interaction effect of irrigation regimes and intercropping system on weed density (a) and weed biomass (b) by two years. Different letters indicate a significant difference (LSD’s significant difference test, P≤0.05). Planting ratios of saffron:chickpea, (C1) saffron mono-crop, (C2) chick­­­­­­­­pea mono-crop, (C3) 1saffron:1chickpea, (C4) 2saffron:2chickpea, (C5) 2saffron:1chickpea, and (C6) 3saffron:1chickpea, respectively.

**Fig. 4.** Bar plots of mean values of both, the (Shannon index) (a), Pielou index (b), and species richness (c) for the irrigation regimes. Different letters indicate a significant difference (LSD’s significant difference test, P≤0.05).

**Fig. 5.** Bar plots of mean values of both, e(Shannon index) (a), Pielou index (b) and species richness (c) for the intercropping systems in 2019 and 2020 years. Different letters indicate a significant difference (LSD’s significant difference test, P≤0.05). Planting ratios of saffron:chickpea (C1) saffron mono-crop, (C2) chick­­­­­­­­pea mono-crop, (C3) 1saffron:1chickpea, (C4) 2saffron:2chickpea, (C5) 2saffron:1chickpea, and (C6) 3saffron:1chickpea, respectively.

**Fig. 6.** Bar plots of mean values of both the (Shannon index) (a) and species richness (b) for the year effect. Different letters indicate a significant difference (LSD’s significant difference test, P≤0.05).
