# Supplementary material for: Weed community changes in saffron+chickpea intercropping under different irrigation management
Source: PLoS One. 2023 May 26;18(5):e0286474. doi: 10.1371/journal.pone.0286474 (PMC10218734; doi:10.1371/journal.pone.0286474)
Supplement: Attachment — Submitted filename: Response to the reviewers comment.docx [file pone.0286474.s005.docx]

**Dear Dr. ....,**

We would like to thank you for the letter, and the opportunity to submit a revised copy of this manuscript. We would also like to take this opportunity to express our thanks to the reviewers for the comments. We believe have resulted in an improved revised manuscript, which you will find uploaded alongside this document. The manuscript has been revised to address the reviewer's comments, detailed in your letter properly as explained below.

Reviewer #1:

1. Full and short title are same.

- In was edited to "Weed response to irrigation in saffron+chickpea intercropping".

2. Nutrient management part is still missing in material & method section.

- It was added in lines 128-130.

3. Line 85: 27% and 15%.

- It was corrected.

4. Line 89: Correct sentence.

- It was corrected.

5. Line 89: Write scientific name in italic.

- Thank you, the text was edited.

6. Line 93: Remove according to.

- It was corrected.

7. Line 95: Saffron.

- It was corrected.

8. Line 99: Saffron+chickpea

- It was corrected.

9. Line 114: Correct alignment.

- It was corrected.

10. Line 116: m2 in superscript

- It was corrected.

11. Line 121: Replace data with date.

- It was corrected.

12. Line 125: Give the stages when you applied irrigations.

- It was presented in table 2.

13. Line129: figure.

- It was corrected.

14. Line171: Use multiplication symbol instead of *.

- It was corrected.

15. Line191: Fig 3a & 3b.

- It was edited and corrected.

16. Line Discussion part need to improve.

- It was corrected.

17. Yield data is missing.

- It was added the text. Please see in 279-288 lines.

Reviewer #2:

a. The authors have done sufficient revisions. However, the minor English language related issues and formatting of references as per the journal format may be done.

- I really appreciate your nice comment. The reference was formatted based on PLOS format. Also English language related issues was edited.

**Best regards,**

**Corresponding author,**
